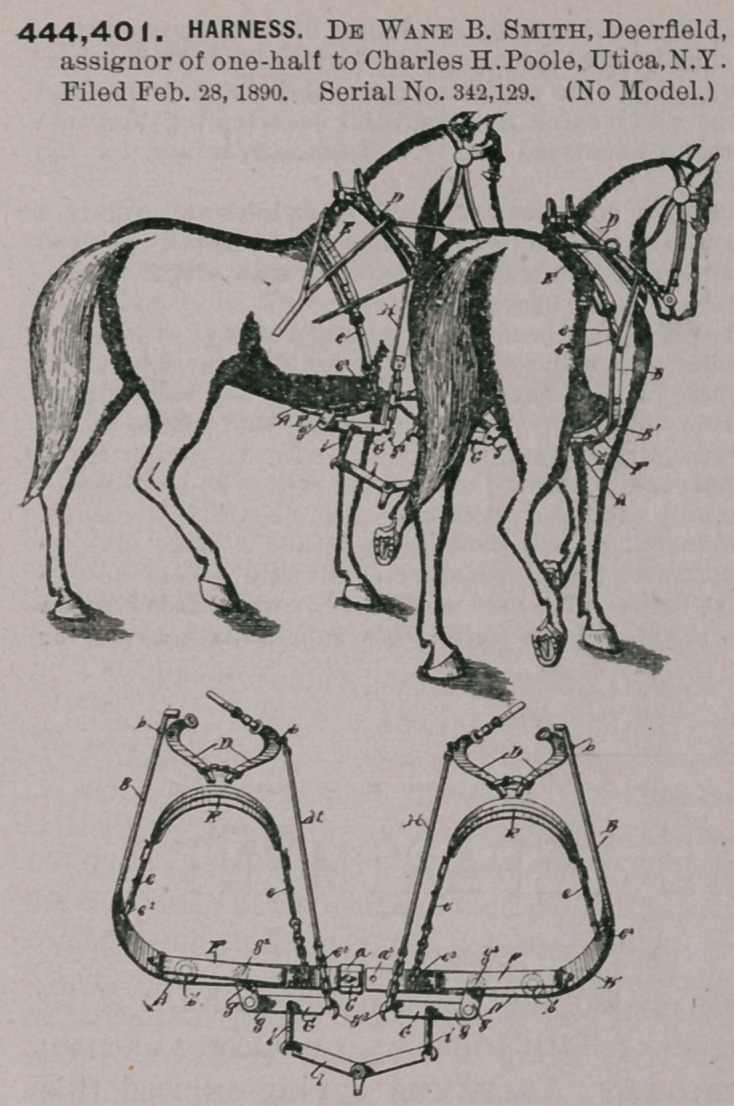# Recent Patents

**Published:** 1891-02

**Authors:** 


### RECENT PATENTS

RELATING TO

### VETERINARY MEDICINE AND ANIMAL INDUSTRY.

*Issued by U. S. Patent Office for Month of January,* 1891.

**444,003. COLLAR-PAD.** FRANCIS M. LIMBAUGH, Woodbridge, Cal. assignor of one-half to Allen T. Covell, same place. Filed Sept. 5, 1890. Serial No. 363,973. (No model.)

*Claim.*—The improved collar-pad herein described, comprising the leather B, slitted to form straps B2, the metal ridge A, having its opposite ends forked and secured at each end to the extended corners of said leather, the free ends of the straps B2 lying upon the opposite corners of the leather, as shown, the straps C, secured midway of their length to the straps B2, and the buckels secured to the legs of the ridge and body-strap, whereby said straps B2 are substained at a lower altitude than the ridge and central portion of the leather substantially as specified.

---

**444,570. SPREADER FOR GAITING HORSES.** JAMES R. PHELPS, Sacramento, Cal. Filed Oct. 2, 1890. Serial No. 366.892. (No model.)

*Claim.*—A spreader for horses, consisting of the curved elastic arms, the lower ends of which have straps connected with them to encircle the hind legs of the horse, and a support upon the harness, in which the upper ends of the arms are swiveled or pivoted to turn, substantially as herein described.

2. A device or spreader for horses, consisting of the elastic curved arms having their upper ends movable independently in a support upon the harness, the lower ends connected with straps to encircle the hind legs of the horse, whereby an outward pull is exerted upon the legs and the elastic arms are allowed to oscillate and move in unison with the movements of the horses legs, substantially as herein described.

3. The independent elastic arms having the lower ends provided with straps to encircle the horse's hind legs, a pad adapted to be placed upon the horse's back and having supports or journals in which the upper ends of the elastic arms turn horizontally so as to move independently of each other, and a means of connecting said support with the girth or surcingle and retaining it in place, substantially as herein described.

4. The curved elastic movable arms supported from a pad upon the horse's back, straps connecting with the lower ends of said arms and adapted to surround the horse's legs, and the extending bar G, by which the outer portion of the strap is kept from contact with the outer side of the leg, substantially as herein described.

5. The combination, with the straps D, suspended from arms connected with the harness, of rigid bars within said straps, having end loops or buckles provided with tongues adapted to enter holes in the straps, whereby the straps are adjusted, substantially as herein described.

**444,023. TOE-WEIGHT AND QUARTER-BOOT FASTENER.** ORLANDO J. SEFTON, Sedgwick, Kans. Filed Apr. 22, 1890. Serial No. 349,035. (No model.)

*Claim.*—1. The combination of a horseshoe having a tapering integral spur provided at its upper end with a loop, an adjustable toe-weight on said spur and having a set-screw, and a strap passing through said loop above the toe-weight, for the purpose set forth.

2. The combination of a horseshoe having a tapering integral spur provided at its upper end with a loop, an adjustable toe-weight on said spur and having a set-screw, and a boot having a fastening-strap passing through said loop above the toe-weight, for the purpose set forth.

**444,032. HORSE-TRAINING HARNESS.** JOHN H. WHITAKER, Davenport, Iowa. Filed Dec. 19, 1889. Serial No. 334,345. (No model.)

*Claim.*—In a horse-training harness, the combination of straps 9 and 10, each having elastic material incorporated therein, the limb straps attached thereto, and the body-straps, having elastic material incorporated therein, attached to such limb-straps, substantially as described.

**444,085. BRIDLE.** HENRY G. BRENT, Brunswick, Mo. Filed July 10, 1890. Serial No. 358,318. (No model.)

*Claim.*—1. The herein described bridle, consisting of a single piece of rope comprising the crown-piece C, an adjustable head stall H, leading from one of the strands of said crown-piece, throat-latch knots K, each comprising another strand of said crown-piece and the return member of the headstall, and the throat-latch T, leading from said knots, substantially as and for the purpose set forth.

2. The herein described bridle, consisting of a single piece of rope comprising a crown-piece coil C, an adjustable headstall H, leading from one of the strands of said crown-piece coil to the bit and returning to throat-latch knots K, a brow-band consisting of a twist W, embracing said headstall and having loops O at its ends, the second strand of said crown-piece coil C entering said side knots K, leading thence through said loops O, and forming the throat-latch T, and the headstall also entering said side knots and leading thence upwardly through a double knot D and into the opposite sides of the brow-band twist W, the whole arranged and adapted for use substantially as herein before set forth.

**444,048. BRIDLE ATTACHMENT.** Andrew Chezem, Sergeant Bluff, Iowa, assignor of one-half to Andrew J. Huntley, same place. Filed Sept. 17, 1889. Serial No. 324,261. (No model.)

*Claim.*—1. As an improved article of manufacture, a coil-spring having an integral buckle-frame at its top end, a tongue attached to the said frame, and an integral ring at its lower end, for the purposes stated.

2. The combination of a coil-spring having a buckle at its top end and a ring at its lower end, the throat-latch of a bridle, and a driving rein, to operate in the manner set forth, for the purposes stated.

**445,000. HORSESHOE.** Henry S. Briscoe, Morrisonville, Ill. Filed Sept. 19, 1890. Serial No. 365,460. (No model.)

*Claim.*—The combination, with a horseshoe having calks on its heels and toe, opposite perforated ears on its side limbs an upwardly-projecting lug on its toe, and transverse ribs on its top face entering mating grooves in the hoof, of a locking-bar bifurcated at its lower end and pivoted thereat to the lug on the shoe and laterally enlarged at the upper end and cross-grooved above said flanged enlargement, an arched clamping-bar having threaded ends passing through the ears on the shoe and provided with nuts, and a set-screw adjustable in the locking-bar near its lower end and provided with a swiveled presser-block having tangs on its lower face, substantially as set forth.

**444,577. FEED-RACK.** John F. Fitzgerald, Trenton, Tenn. Filed Apr. 11, 1890. Serial No. 347,459. (No model.)

*Claim.*—The hereinbefore-specified trough, having notch $h_2$ in its upper edge and having the slots G G, the slot-frame D, pivoted at its rear end to the trough and having the pulley $d_1$ at its front end, the bottom $f$, pivoted at its rear end and having portions projected through the slots G G, and the cord H, connected with the front end of the bottom $f$ and passing over the said pulley $d_1$, and having weight $h$ at its upper end, and the knot $h_1$, substantially as described, for the purpose specified.

**444,324.** **WATERING-TROUGH FOR STOCK.** JOHN ALLIS. Lowville, N. Y. Filed April 25, 1890. Serial No. 349,574. (No model.)

*Claim.*—An appparatus for watering stock, consisting of the main supply-pipe C, with its vertical branches E E, the former provided with the regulating valves B and I and air-valve J, the branch pipes entering into and connected with separate and individual troughs D D, the last of the series being provided with the safety or overflow drainage-pipe H, all arranged as set forth as described.

**444,387.** **DEVICE FOR HOLDING HORSES.** JACOB J. HARRIS, Highland, Ohio. Filed Oct. 9, 1890. Serial No. 367,549. (No model.)

*Claim.*—In a harness attachment for the purpose set forth, the ropes or flexible connections C and D, connected to each other by clamps A and B, one of said clamps carrying a ring, the rope C, forming a loop adjacent to the clamp A, a strap and buckle having a loop through which the flexible connection D passes, the free end of said rope being passed through the ring carried by the clasp B, substantially as set forth.

2. A harness attachment consisting of a flexible connection C, having an end loop and flexible connection D, clamped to each other, as shown, the connection D, extending beyond the clamp B, in combination with the strap F and auxiliary rope G, having a looped end, for the purpose set forth.

**445,050.** **HOOF-PAD.** JOSEPH T. DUCK. Geneva, N. Y. Filed July 31, 1890. Serial No. 360,580. (No model.)

*Claim.*—1. The combination, witn the shoe and the lips projecting inwardly from the rear ends thereof, of a pad secured to the front end thereof and engaging with said lips when the pad is under pressure, and lateral arms upon the pad, arching outward and having their free ends in contact with the lower face of the shoe.

2. The combination, with the shoe and the lips projecting inwardly from the rear ends thereof, of a pad secured to the front end thereof and engaging with said lips when the pad is under pressure, and lateral arms upon the pad, arching outward. with their free ends in contact with the lower face of the shoe, and shoulders upon the arms engaging with the inner edges of the shoe when pressure is applied to the pad.

**444,425. BIT FOR HORSES.** CHARLES P. GREGORY, Stillwater, Minn. Filed Aug. 7, 1890. Serial No. 361,337. (No model.)

*Claim.*—In a bridle-bit, the main bit A, an overcheck-bit D1, having a central loop D2 and connected to said main bit by a flexible joint, the central or pivotal point of said joint being between said overcheck-bit and main bit, whereby any downward movement of the horse's head will cause said overcheck-bit to be oscillated upon said flexible joint and cause said loop to be turned upward, but without materially altering the relative positions of the parts A D1, substantially as and for the purpose set forth.

**444,428. COW-TAIL HOLDER.** EDWIN G. FARNHAM, Dover, Me. Filed Feb. 24, 1890. Serial No. 341,602. (No model.)

*Claim.*—In a cow-tail holder, the combination of the casing-plates, a bar secured between the same and having a jaw and a curved arm extending laterally in opposite directions, a jaw mounted pivotally at the corner opposite to the fixed jaw and having a laterally-extending handle, a curved arm mounted pivotally at the corner opposite to the fixed arm and having a laterally-extending handle, the lugs extending from said handles past each other, and a spring arranged within the casing and bearing against the inner lug, substantially as and for the purpose set forth.

**444,561. POULTRY-CRATE.** ROBERT G. THOMASSON, Bumpass, Va. Filed Oct. 9, 1890. Serial No. 367,546. (No model.)

*Claim.*—1. The chicken coop or crate comprising the bottom having the side and end projecting portions forming the normally upturned portions or sides and ends of the coop connected together by the short tuck-in corner pieces or strips, and the inner and outer opposite strips or pieces lapping and secured to the top edge strips of the sides and suitably re-enforced thereat, substantially as and for the purpose set forth.

2. The chicken-coop consisting of the bottom wicker-work portion having side end portions held together as described, and having upper and lower bottom re-enforcing pieces or strips, also re-enforcing metallic pieces or strips near their corner edges, the wire netting covered bows with their braces or stays, said bows being secured to said bottom portion, the upright stays or braces secured to said bottom portion, and the end bows and the re-enforcing metal pieces secured to said upright stays and to said bottom portion and to the central top brace of said bows and the central underneath strip or brace of said bottom portion, substantially as set forth.

**444,975. CHECKREIN FOR HARNESS.** WILLIAM W. DAVISSON, Mineral Point, Wis. Filed Sept. 9, 1890. Serial No. 364,397. (No model.)

*Claim.*—. The combination of a check rein folded or doubled upon itself, forming the loop 7, and having means, as a buckle, for rigidly connecting the free end of the strap with the body thereof, the stationary band or sleeve arranged exteriorly on said loop, the bail adapted to be connected to a check-hook and rigid with the sleeve or band, a sliding plate arranged within the loop 7 and guided by the bail, and the cushion-spring housed within the loop 7 and operating between the sliding plate and the bail, substantially as and for the purpose described.

2. The combination of a checkrein folded or doubled upon itself and having its free end rigidly secured to the body of the strap, as by a buckle, to form the loop 7, the stationary sleeve or band fitted on said loop of the strap, the longitudinal bail rigid with the sleeve or band and having one end terminating within the loop of the strap and its other end provided with an eye 10, adapted to engage a checkhook the notched plate arranged within the loop of the strap and fitted between the sides of the bail, the longitudinal rod attached to the sliding plate, and the coiled spring fitted around said rod and operating between the sliding plate and the inner end of the bail, substantially as described.

---

**444,911. AUTOMATIC STOCK-WATERING TROUGH.** CHARLES A. YONT, Brock, Nebr. Filed May 21, 1890. Serial No. 352,673. (No model.)

*Claim.*—In an automatic stock-watering trough, an actuating-bail encircling the upper edge of the drinking-cup, fulcrumed at the rear, the ends there forming a vertically-depending leg resting normally in operative contact with the projecting head of the valve-bolt and adapted to admit water to the cup when bail is depressed by the chin or neck of the animal, substantially as and for the purpose stated.

**444,677. INDEPENDENT CHECK FOR HORSES.** CARLO R. TAYLOR, Berlin, Wis., assignor of one-half to Josiah T. Whitcomb, same place. Filed Nov. 19, 1889. Serial No. 330,921. (No model.)

*Claim.*—A chin-plate A, notched at its upper end, having an orifice at its lower extremity, and having attached thereto the spur-plate B, provided with spurs, the spring D, and rein-lugs E, substantially as described.

**444,993. HALTER.** LOUIS E. SHIPPY, Sandy Hill, N. Y. Filed Oct. 29, 1890. Serial No. 369,664. (No model.)

*Claim.*—A halter comprising a head-band, a throat-latch strap connected with the ends of the head-band, the check-guards C C1, having two slits *a b*, the check-straps A A1, passing down through the brow-band and loosely through the slits *a b* to form the nose-band B, the loose rings D D1, suspended on the loops *e*, formed by the portions of the straps A A1 where they cross the guards C C1 between the slits *a b*, and the curb-strap G, connecting the two rings D D1, substantially as set forth.

**444,648. MARTINGALE ATTACHMENT FOR HARNESS.** STILLMAN E. MATHEWS, Fullerville, N. Y. Filed Sept. 25, 1890. Serial No. 366,108. (No model.)

*Claim.*—1. A martingale attachment for harness, comprising a rigid rod which is forked at one end, has a bit-bar across the limbs of the fork that may be connected to a bridle, and a sleeve which slides on the rod, is securable thereon, and attachable to the breast collar or a strap of the harness, substantially as set forth.

2. A rigid martingale for harness, comprised, essentially of a bifurcated rigid rod having a bridle bit-bar attached to the limbs of its bifurcated portion, a knuckle joint on the rod near the bifurcated end, and a rigid sleeve that is adapted to slide on the rod and be secured thereon at any point of longitudinal adjustment and also attachable to the breast-collar or strap of the harness, substantially as set forth.

**444,761. HARNESS-SADDLE.** WILLIAM O. MILLER, Quincy, Ill. Filed May 19, 1890. Serial No. 352,375. (No model.)

*Claim.*—1. In a harness-tree, the combination, with the check-hook plate provided with loops, eyes, or slots as described, of the metal side plates provided with the hooks and terrets passed through said hooks, substantially as described.

2. In a harness-tree, the combination, with the check-hook plate provided with loops, eyes, or slots as described, of of the metal side plates having the hooks the bridge pieces beneath the hooks, and the terrets passing through the hooks and said bridge-pieces, substantially as described.

3. The side plate provided with a hook, as described, and made open or cut away beneath the hock, in combination with the shouldered bridge-piece located beneath the end of the hook and seated in the opening in the plate and with the terret, substantially as described.

**444,401. HARNESS.** DE WANE B. SMITH, Deerfield, assignor of one-half to Charles H. Poole, Utica, N.Y. Filed Feb. 28, 1890. Serial No. 342,129. (No Model.)

*Claim.*—In combination with the traces and saddles of a double harness, the herein-described rigid cross or draw bar connected to said traces and saddles and composed of sparable divisions having their inner extremities approximated, and a movable catch for engaging said extremities and removably securing them together, whereby a team may be harnessed singly, substantially as and for the purpose specified.

2. In combination with the traces and saddles of a double harness, the herein-described cross or draw bar connected to said traces and saddles and composed of separable divisions having their inner extremities approximated, and a spring-actuated sliping rod secured to one of said divisions and removably engaging the other for removably securing together said divisions, whereby a team may be harnessed singly, substantially as and for the purpose set forth.

3. In combination with the traces and saddles of a double harness, the herein-described cross or draw bar connected to said traces and saddles and composed of separable divisions, a socket provided on one of said divisions and adapted to receive the end of the other division, and a catch for removably securing said parts together, substantially as and for the purpose specified.

4. In combination with the traces and saddles of a double harness, a metallic cross or draw bar cennected to said traces and saddles and adapted to be placed beneath the horse, and a strap above said metallic cross or draw bar adapted to be imposed between the horse and the said bar and having its opposite extremities secured to said cross-bar, substantially as and for the purpose specified.

5. In combination with the traces and saddles of a double harness, a metallic cross or draw bar connected to said traces and saddles and composed of separable divisions having their inner extremities removably secured together, whereby a team may be harnessed singly, and a strap above said cross or draw bar adapted to be imposed between the horse and said bar, substantially as and for the purpose set forth.

6. In a harness, the combination, with the collars, of a double harness, metallic traces loosely connected at their forward extremities to said collars, a cross or draw bar loosely pivoted to the rearward extrcmities of said traces, and a saddle for supporting said cross bar, substantially as specified.

7. In a harness, the combination. with a collar, a cross-bar, and a saddle for supporting said cross-bar, of a metallic trace having its forward end connected to said collar, and having its rearward end deflected below the plane of its forward end, and latterally-extending arm provided on said rearward end and connected to said cross-bar, substantially as specified.

8. In combination with the collars and saddles of a double harness, the combination of metallic traces loosely connected at their forward extremities to said collars, a cross or draw bar loosely connected to the opposite extremities of said traces and composed of separable divisions, and a catch for removably securing together said divisions whereby a team may be harntssed singly, substancially as and for the purpose set forth.

9. The herein-described harness, composed of a pair of saddles and collars, a cross or draw bar connected to the saddles, outer metallic traces pivotally secured to said collars and cross bar, single whiffle-tree connected to said single whiffle-trees, substantially as and for the purpose specified.

10. The herein-described harness, composed of a pair of a pair of collars and saddles, a cross or draw connected with said saddles, outer metallic traces pivotally connected to said cross bar, single whiffletrees, links connecting the said whiffletrees and cross-bar, and a double whiffletree connecting to the said single whiffltrees, substantially as and for the purpose set forth.

11. The herein described harness, composed of a pair of collars and saddles, a cross or draw bar connected to said saddles and composed of separable divisions, and a catch for removably connecting said divisions, whereby a team may be harnessed singly, outer metallic traces pivotally connected to said collars and to said bar, single wiffletrees, links between the said whiffletrees and cross bar, and a double whiffletree connected to the said single whiffletrees, substantially as and for the purpose set forth.

## Figures and Tables

**Figure f1:**
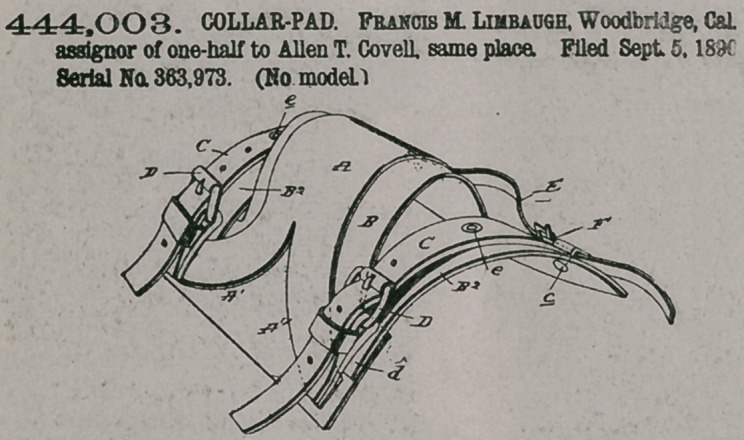


**Figure f2:**
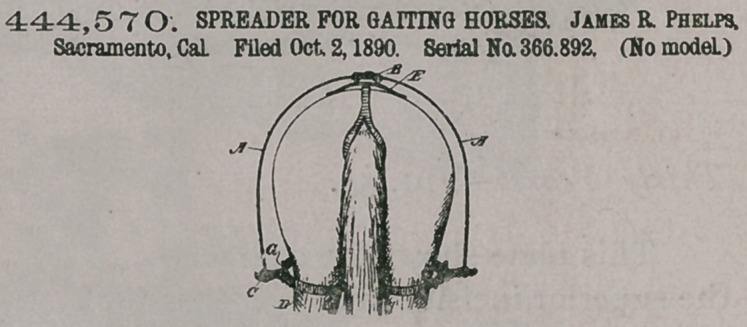


**Figure f3:**
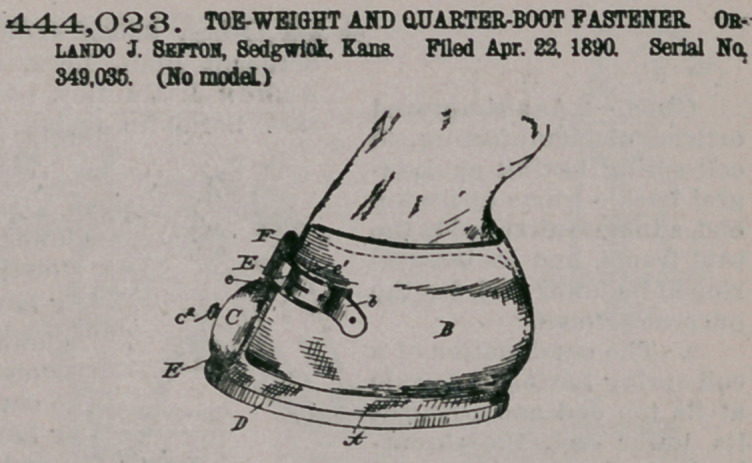


**Figure f4:**
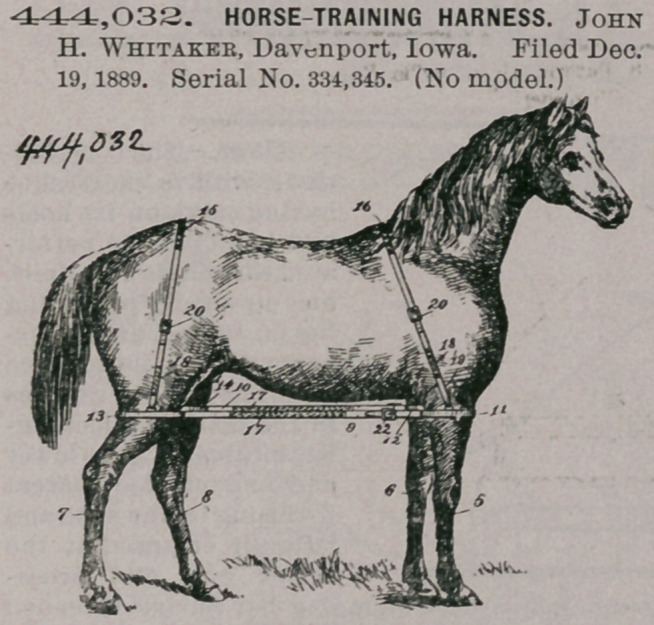


**Figure f5:**
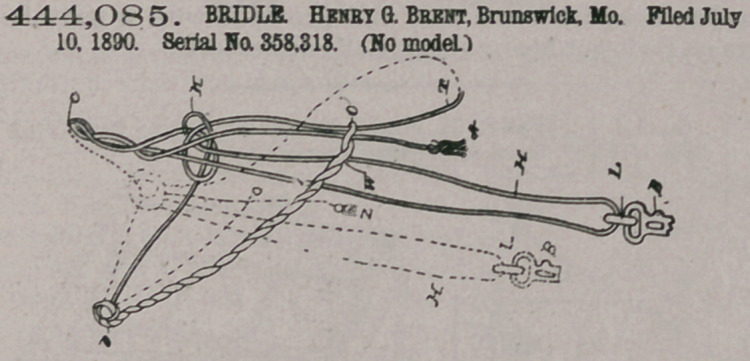


**Figure f6:**
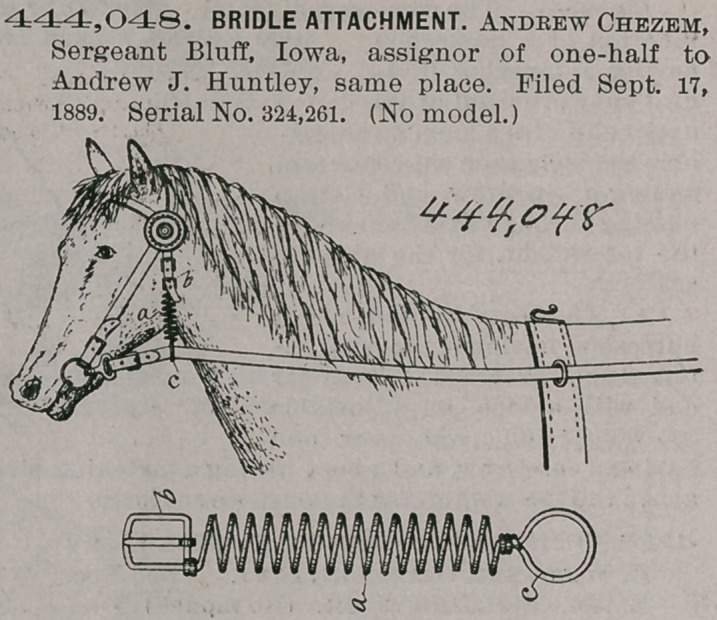


**Figure f7:**
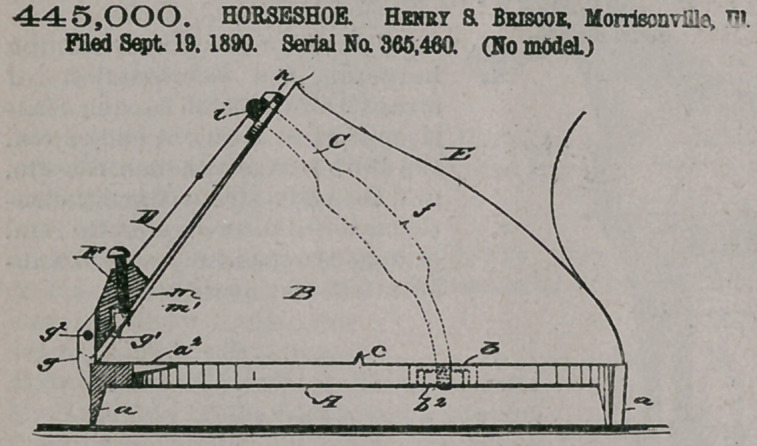


**Figure f8:**
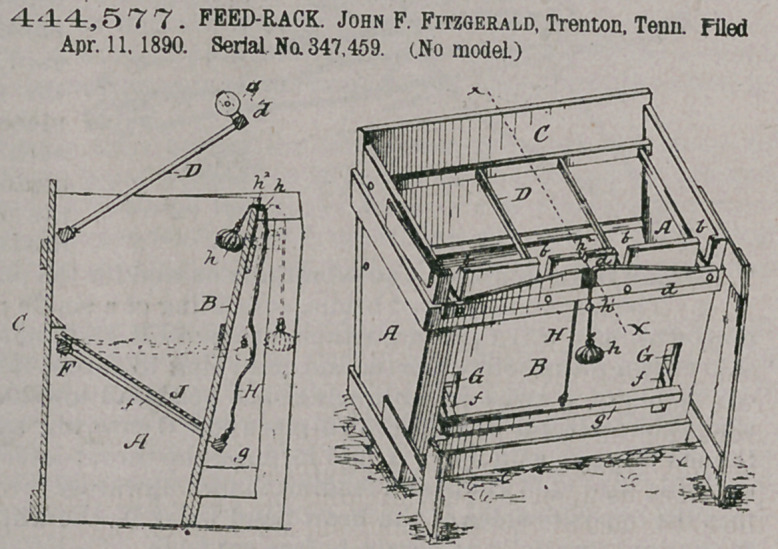


**Figure f9:**
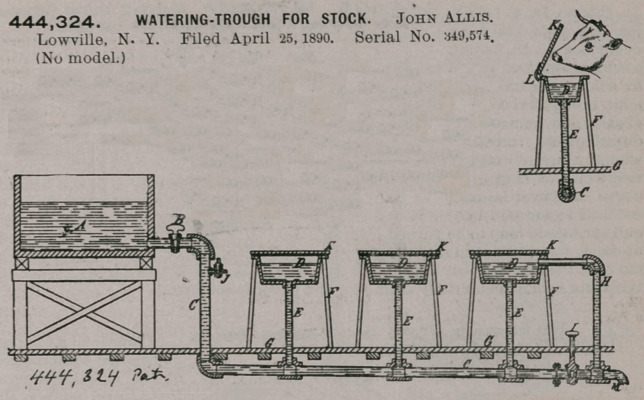


**Figure f10:**
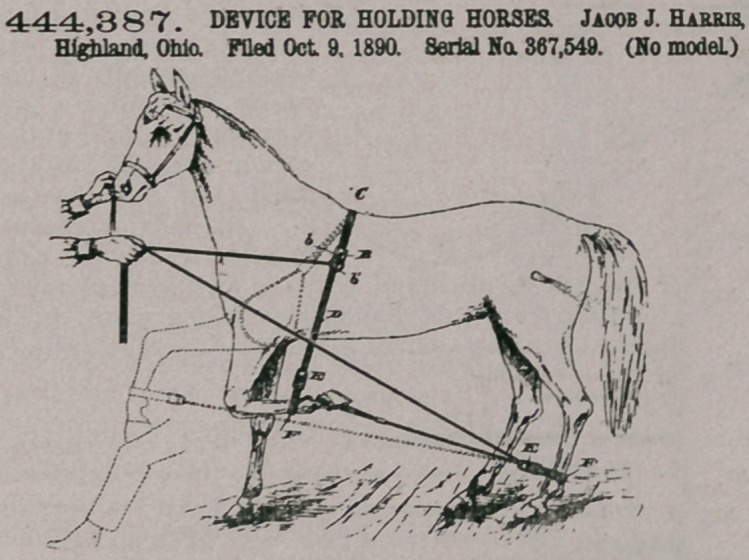


**Figure f11:**
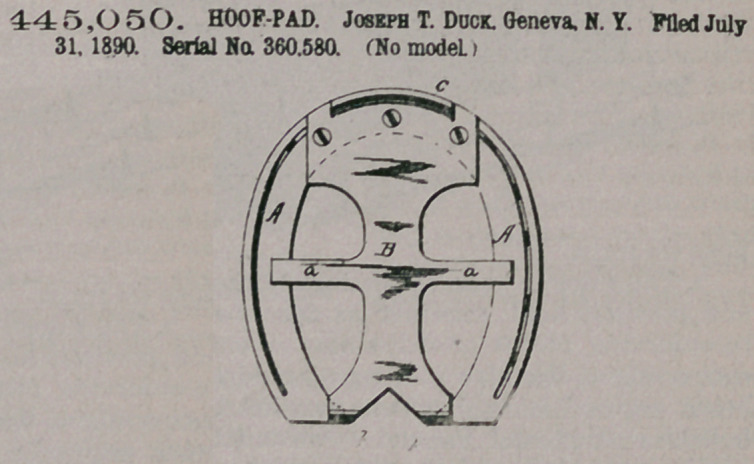


**Figure f12:**
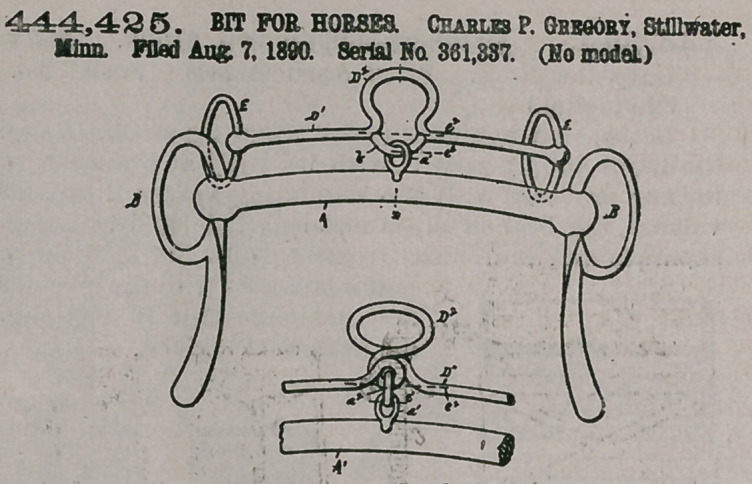


**Figure f13:**
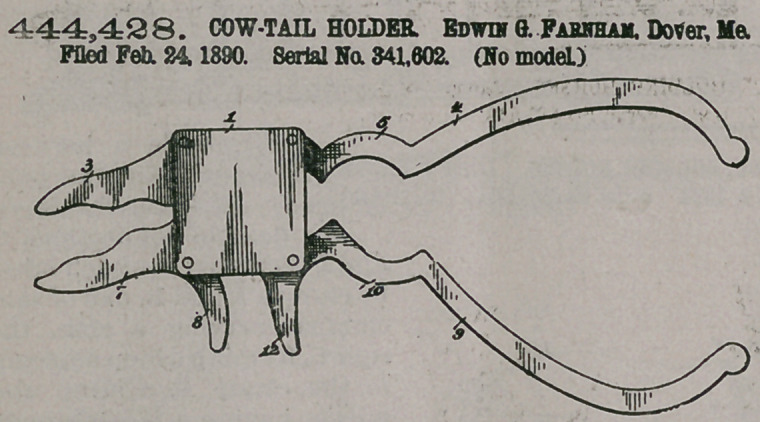


**Figure f14:**
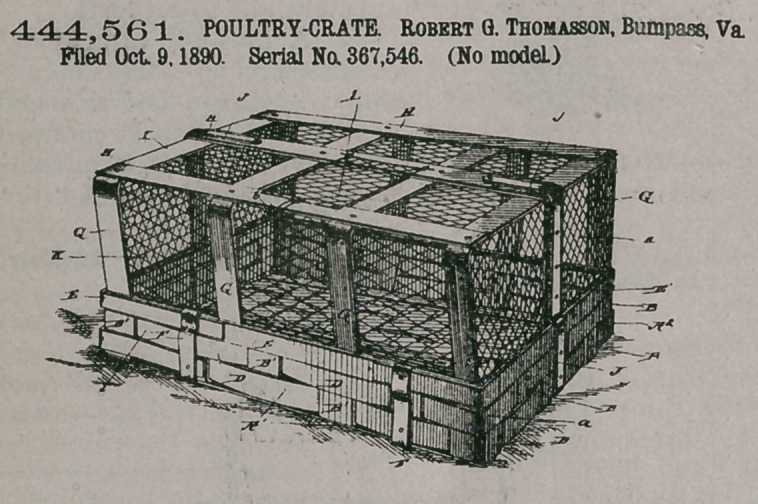


**Figure f15:**
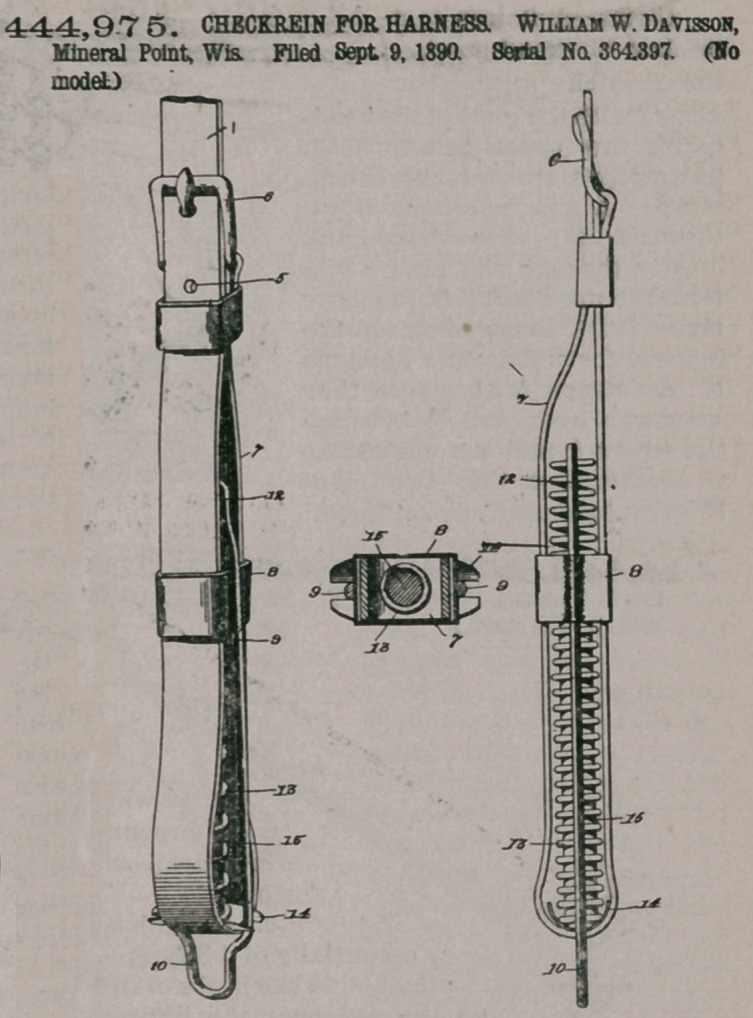


**Figure f16:**
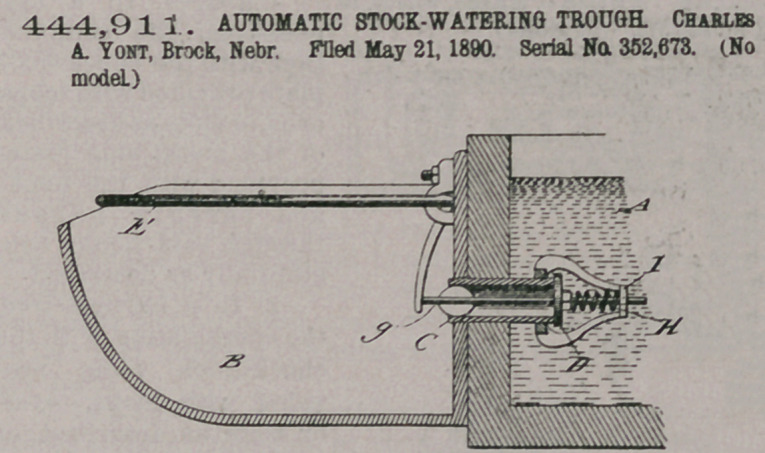


**Figure f17:**
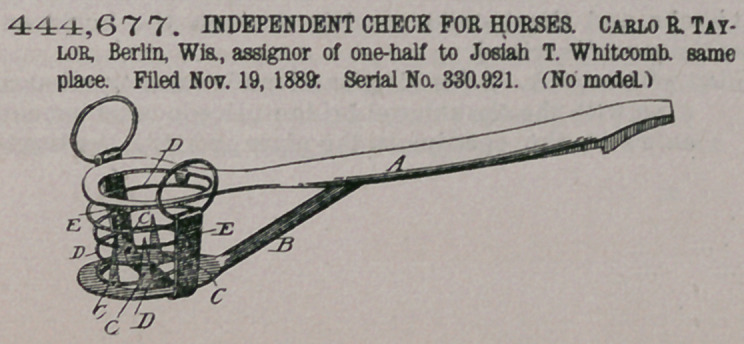


**Figure f18:**
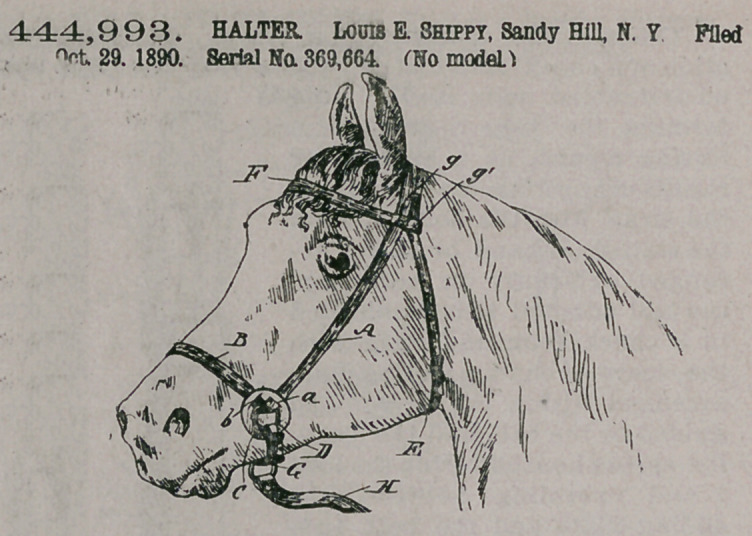


**Figure f19:**
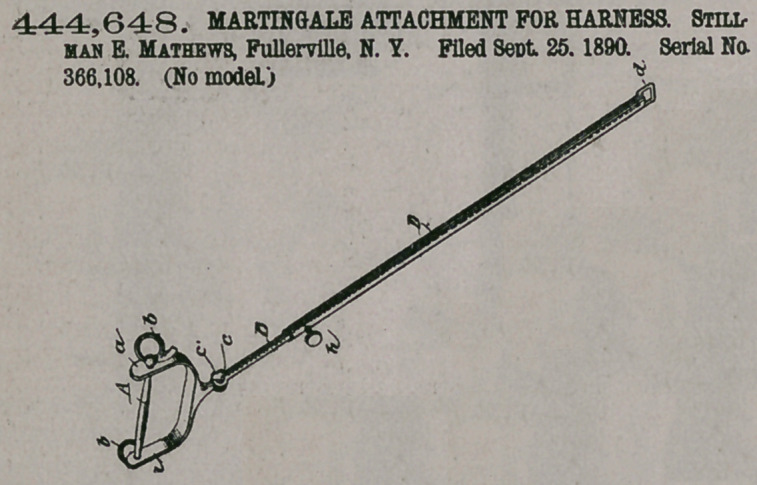


**Figure f20:**
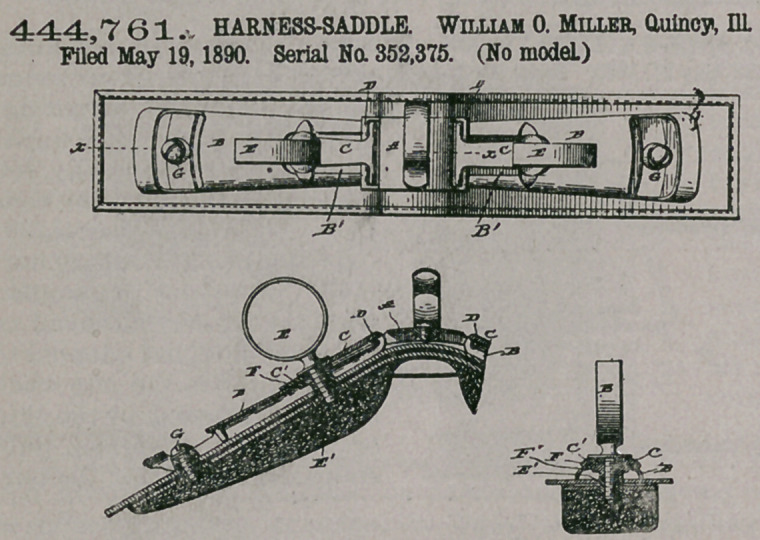


**Figure f21:**